# Characterization of molecular diversity and genome-wide association study of stripe rust resistance at the adult plant stage in Northern Chinese wheat landraces

**DOI:** 10.1186/s12863-019-0736-x

**Published:** 2019-03-26

**Authors:** Fangjie Yao, Xuemei Zhang, Xueling Ye, Jian Li, Li Long, Can Yu, Jing Li, Yuqi Wang, Yu Wu, Jirui Wang, Qiantao Jiang, Wei Li, Jian Ma, Yuming Wei, Youliang Zheng, Guoyue Chen

**Affiliations:** 1 Triticeae Research Institute, Sichuan Agricultural University, Wenjiang, Chengdu, Sichuan 611130 People’s Republic of China; 2 State Key Laboratory of Crop Genetics of Disease Resistance and Disease Control, Sichuan Agricultural University, Wenjiang, Chengdu, Sichuan 611130 People’s Republic of China; 3 College of Agronomy, Sichuan Agricultural University, Wenjiang, Chengdu, Sichuan 611130 People’s Republic of China

**Keywords:** Wheat, Landrace, Stripe rust, Genome-wide association study, Diversity arrays technology, Simple sequence repeat

## Abstract

**Background:**

Stripe rust is a serious fungal disease of wheat (*Triticum aestivum* L.) caused by *Puccinia striiformis* f. sp. *tritici* (*Pst*), which results in yield reduction and decreased grain quality. Breeding for genetic resistance to stripe rust is the most cost-effective method to control the disease. In the present study, a genome-wide association study (GWAS) was conducted to identify markers linked to stripe rust resistance genes (or loci) in 93 Northern Chinese wheat landraces, using Diversity Arrays Technology (DArT) and simple sequence repeat (SSR) molecular marker technology based on phenotypic data from two field locations over two growing seasons in China.

**Results:**

Seventeen accessions were verified to display stable and high levels of adult plant resistance (APR) to stripe rust via multi-environment field assessments. Significant correlations among environments and high heritability were observed for stripe rust infection type (IT) and disease severity (DS). Using mixed linear models (MLM) for the GWAS, a total of 32 significantly associated loci (*P* < 0.001) were detected. In combination with the linkage disequilibrium (LD) decay distance (6.4 cM), 25 quantitative trait loci (QTL) were identified. Based on the integrated map of previously reported genes and QTL, six QTL located on chromosomes 4A, 6A and 7D were mapped far from resistance regions identified previously, and represent potentially novel stripe rust resistance loci at the adult plant stage.

**Conclusions:**

The present findings demonstrated that identification of genes or loci linked to significant markers in wheat by GWAS is feasible. Seventeen elite accessions conferred with stable and high resistance to stripe rust, and six putative newly detected APR loci were identified among the 93 Northern Chinese wheat landraces. The results illustrate the potential for acceleration of molecular breeding of wheat, and also provide novel sources of stripe rust resistance with potential utility in the breeding of improved wheat cultivars.

**Electronic supplementary material:**

The online version of this article (10.1186/s12863-019-0736-x) contains supplementary material, which is available to authorized users.

## Background

Stripe rust caused by *Pst* is also known as yellow rust because of the spore color during its asexual infection cycle on wheat [1]. Stripe rust is a serious disease of wheat worldwide that mainly damages leaf tissues. Stripe rust significantly reduces wheat yield by at least 10%, and up to 100% under severe infections [2]. The stripe rust fungus has diversified into a large number of races possessing different combinations of virulence genes. These races have the capability of circumventing the host resistance genes, and in combination with their capacity for long-distance dispersal, subsequently creating the potential for destructive epidemics in susceptible varieties under favorable conditions [3, 4]. In China, the most severe epidemics of wheat stripe rust occurred in 1950, 1964, 1990, and 2002, and caused substantial yield losses of wheat, which were estimated at 6.00, 3.20, 2.65 and 1.40 million metric tons, respectively. In 2017, a stripe rust epidemic affected 1.65 million hectares in 12 provinces [4, 5]. Application of fungicides is widely used in the control of stripe rust, however, this practice adds considerable cost to wheat production. In contrast, growing resistant cultivars is considered to be the most effective, environment-, and consumer-friendly means to manage stripe rust [2, 6–8].

Resistance to stripe rust can be classified into two types, on the basis of the growth stage of resistance expression: seedling/all stage resistance (ASR) and APR [2, 9, 10]. The ASR is effective at seedling and adult plant stages and is usually race specific and qualitatively inherited, but it can be overcome by new races of the pathogen. In contrast, APR is only effective at adult plant stages when warm weather is prevalent, and is usually non-race specific and quantitatively inherited, and more likely to be durable [11]. Previously, 80 *Yr* genes for stripe rust resistance have been identified and formally named [12, 13], however, the majority of these resistance genes are ineffective against new *Pst* races [14–16]. Therefore, identification of novel sources of resistance for deployment in breeding programs is a matter of urgency.

Wheat landraces are traditional varieties that were selected by farmers in the field for preferable agronomic traits, but concurrently were also indirectly selected for disease resistance [17]. As the included resources in the primary gene pool, wheat landraces harbor many novel and stable resistance genes that can be utilized for the improvement of modern high-yielding cultivars [18]. The landraces carry homologous chromosomes that readily recombine with those of hexaploid wheat [19]. Wheat landraces are regarded as untapped genetic resources with potentially useful genetic diversity in view of their limited use in modern breeding programs. The utilization of wheat landraces as a valuable source of disease resistance has been demonstrated previously [20]. Numerous QTL for stripe rust resistance have been identified in recent decades [21]. The usual method for identification of QTL is traditional QTL mapping, also known as linkage mapping. The technique is applied to identify underlying genetic variations that co-segregate with a trait of interest using a bi-parental mapping population [22]. However, QTL mapping is fundamentally limited to the comparatively low allelic diversity of the two parents used for a cross and low recombination events which impair the mapping resolution [23]. Alternatively, GWAS has been used successfully in mapping QTL in different species, such as rice [24], barley [25], maize [26], soybean [27], cotton [28], oat [29], secale [30], eggplant [31], tomato [32], perennial ryegrass [33], chickpea [34], grape [35], sugarcane [36] and *Brassica napus* [37]. In addition, GWAS has been applied to study diverse traits in wheat, such as rust resistance [3], abiotic stress [38], yield-related traits [39] and agronomic traits [40]. Thus, GWAS is proven to be an appropriate approach for identification of novel genetic loci.

In this study, we evaluated 93 wheat landraces grown in the Northern Chinese wheat-growing zone (I-Northern Winter Wheat Zone and VII-Northern Spring Wheat Zone) [41] for resistance to *Pst*. The accessions were evaluated at the adult plant stage using a mixture of *Pst* races prevalent in China over 2 years in two field locations. We identified 32 high-confidence associations and further compared their chromosomal locations with previously mapped *Pst* resistance genes and QTL on the integrated map. The identified loci are suitable for marker-assisted selection (MAS) and further genetic dissection.

## Results

### Adult-stage responses to stripe rust and estimation of heritability

**Fig. 1:**
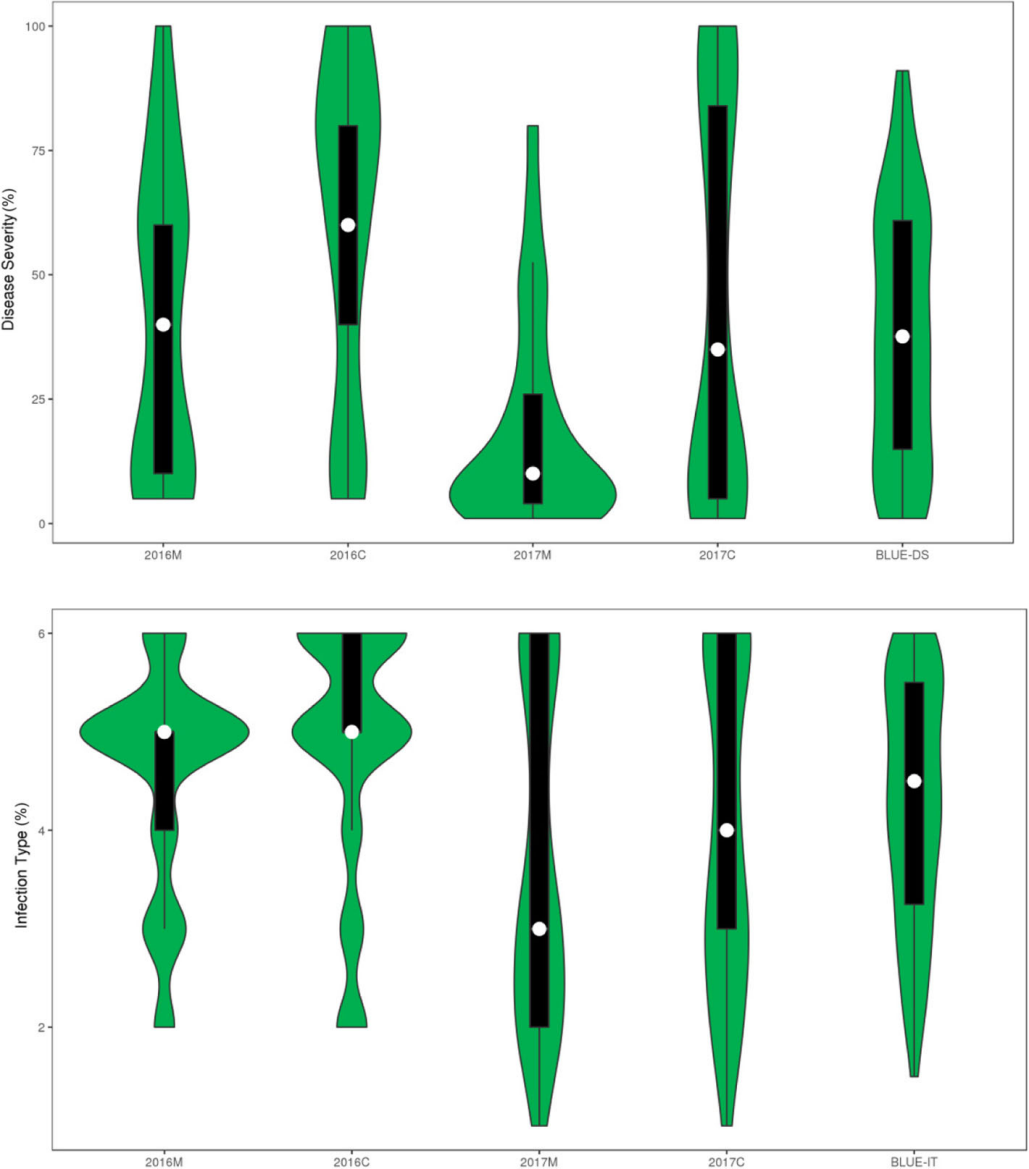
Violin plots illustrating the density distribution of stripe rust response in four environments and BLUE_ALL. The IT data for 2016M, 2016C, 2017M and 2017C were converted from 0, 0; 1, 2, 3 and 4 to 1, 2, 3, 4, 5 and 6 scale, respectively, to allow comparison across all data sets. The white dot displays the median, the top and bottom of the thick black vertical bars represent first and third quartiles, respectively, and the green fill shows DS and IT estimates (*n* = 93). The two graphs were drawn using the omicshare online tool violin2 (http://www.omicshare.com/tools/Home/Soft/violin2)

In the field, we recorded the stripe rust response of the 93 wheat landraces grown in four environments at Mianyang and Chongzhou in 2016 and 2017 (Additional file 1). The landraces displayed diverse adult plant disease responses to a mixture of races prevalent in China. Under each of the four environments and the best linear unbiased estimates (BLUE) for all environments (BLUE_ALL), the phenotypic performance of the 93 landraces varied from 1 to the maximum of 6 in IT, and from 0 to 100% in DS (Fig. 1, Additional file 1). In 2016 at Mianyang, 59.3 and 29.7% of the evaluated 93 landraces showed resistance on the basis of DS and IT, respectively, while in 2016 at Chongzhou, the proportion of resistances among all landraces was 34.2 and 25.3% based on DS and IT, respectively. Proportions 88.2 and 63.4% (in 2017 at Mianyang) and 57.0 and 57.6% (in 2017 at Chongzhong) were recorded in the other two environments (Additional file 1). Among the 93 tested landraces, 17 accessions displayed stable resistance with low values of IT and DS (IT < 4, DS < 60%) under the four artificial inoculation environments. Eight of these accessions were collected from I-zone, namely Hongyucao (Shaanxi), Huangwumanglaomai (Shaanxi), Xiaoqingmang (Gansu), Baimangmai (Gansu), Dabaimai (Gansu), Shanxibaimai (Gansu), Cantiaomai (Shaanxi) and Siqiangxiaomai (Shaanxi). The other nine accessions with stable resistance were collected from VII-zone, namely Mangmai (Inner Mongolia), Dahongpao (Inner Mongolia), Dahongmai (Shanxi), Shishoumai (Inner Mongolia), Xiaohongmai (Inner Mongolia), Hongsesui (Inner Mongolia), Dahongpao (Inner Mongolia), Hongmangmai (Beijing) and Xiaobaisui (Inner Mongolia). The remaining 76 accessions showed higher IT and DS values in one environment, while showing lower values of IT and DS in another environment. Variation in the prevalent pathogen races in the different trials and interactions between genotype and environment may lead to differences in the numbers of resistant accessions across environments. Despite these differences, we observed high correlations coefficients (0.478–0.958, *P* < 0.001) between IT and DS values recorded in the different environments (Additional file 2). These strong correlations were mainly attributed to the similar *Pst* populations that we inoculated in the two planting areas. Broad-sense heritability (*H*^*2*^) for both DS and IT were high across environments, with values of 0.80 and 0.85, respectively (Table 1).

**Table 1:** Summary of stripe rust responses in four environments and BLUE_ALL

	2016 M		2016C		2017 M		2017C		BLUE_ALL	
	IT (1–6)	DS (%)	IT (1–6)	DS (%)	IT (1–6)	DS (%)	IT (1–6)	DS (%)	IT (1–6)	DS (%)
Minimum	2	0	2	0	1	0	1	0	1.50	0.00
Average	4.57	37.91	4.82	58.67	3.73	13.21	4.05	40.90	4.29	36.43
Maximum	6	100	6	100	6	80	6	100	6.00	91.00
Stdev^a^	1.11	31.16	1.29	33.67	1.69	19.40	1.62	39.75	1.21	25.82
CV^b^	0.24	0.82	0.27	0.57	0.45	1.47	0.40	0.97	0.28	0.71
σ_G_^2c^									7.47^**e^	6.83^**^
*H* ^*2*d^									0.85	0.80

^a^ Stdev = Standard Deviation

^b^ CV = Coefficient of Variation

^c^ σ_G_^2^ = Estimate of genotypic variance

^d^ *H*^*2*^ = Heritability

^e^ **P < 0.001

### Population structure and genetic diversity

**Fig. 2:**
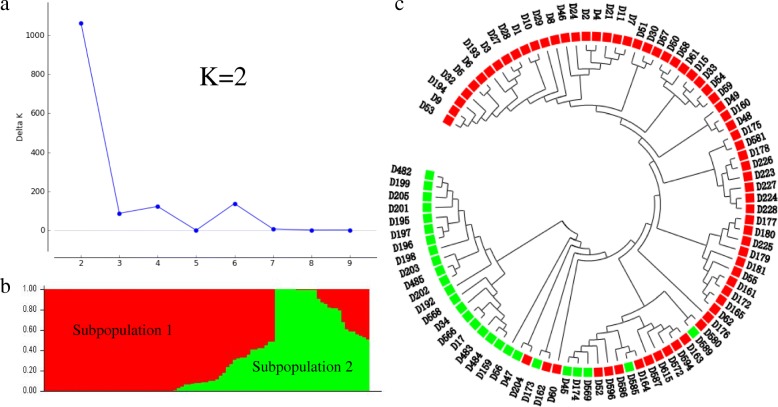
Model-based population structure of the 93 Northern Chinese wheat landraces combined with markers. (**a**) The result obtained from Structure Harvester analysis (k = 2); (**b**) Population structure of the wheat gene pool based on Bayesian inference among 7899 DArT and SSR polymorphism markers; (**c**) Cluster analysis was based on the neighbor-joining algorithm. The red block indicates the 66 accessions varieties of subpopulation 1 and the green block indicates the 27 accessions varieties of subpopulation 2 (Additional file 1)

The optimal number of subpopulation in the 93 wheat landraces panel was determined to be two based on calculation with the STRUCTURE software using a Bayesian clustering model [42] and subsequent application of STRUCTURE HARVESTER (http://taylor0.biology.ucla.edu/structureHarvester/) [43] (Fig. 2a, b). Subpopulation 1 contained 66 accessions, whereas subpopulation 2 contained 27 accessions (Additional file 1). Similarly, construction of a distance-based neighbor-joining tree resulted in a dendrogram in which clustering of the accessions was consistent with the STRUCTURE analysis (Fig. 2c).

The 78 accessions from I-zone were divided into two groups, sixty-four accessions were classified in subpopulation 1 and accounted for 97% of all accessions in subpopulation 1, whereas 14 accessions were classified in subpopulation 2 and accounted for 52% of all accessions in subpopulation 2. Among the 15 accessions from VII-zone included in the study, 13 accessions were grouped in subpopulation 2, accounting for 48% of all accessions in subpopulation 2. Two accessions from VII-zone were grouped in subpopulation 1, comprising 3% of the accessions in subpopulation 1.

In a summary, winter wheat accessions comprised the dominant proportion of subpopulation 1, whereas subpopulation 2 consisted of winter wheat and spring wheat accessions in similar proportions (Additional file 1).

Accessions of similar geographic origins were mixed among marker-based clusters. Accessions in subpopulation 1 consisted of samples collected from Beijing (12%), Gansu (9%), Hebei (14%), Inner Mongolia (2%), Liaoning (3%), Shaanxi (32%), Shanxi (23%), and Tianjin (5%). Accessions in subpopulation 2 contained landraces collected from Hebei (7%), Inner Mongolia (40%), Liaoning (4%), Shaanxi (30%), and Shanxi (19%). It was noteworthy that some sampling regions were aggregated into specific marker-based clusters. In addition, 92% of the accessions derived from Inner Mongolia, which belongs to VII-zone were grouped in subpopulation 2 (Additional file 1).

After filtering, 7107 polymorphic DArT markers and 120 SSR markers with 792 polymorphic allele variations were retained. Among the 7899 polymorphic markers, 2577 were located on the A genome chromosomes, 3655 in the B genome, and 1667 in the D genome. The maximum number (831) of polymorphic markers was observed for chromosome 3B and the minimum number (102) for chromosome 4D (Table 2). Genetic diversity was analyzed using the 7899 markers. Gene diversity and polymorphism information content (PIC) of the whole genome ranged from 0.1017 to 0.5000 and from 0.0966 to 0.3750, with averages of 0.3109 and 0.2540, respectively. Minor allele frequencies (MAF) attained a maximum of 0.3750, with an average of 0.2222 (Table 2, Additional file 3). These analyses revealed a highly significant difference between the two subpopulations with regard to gene diversity and PIC values. Both diversity indices were significantly higher in subpopulation 2 in all of the 21 wheat chromosomes (Additional file 4).

**Table 2:** Summary of markers numbers, PIC, gene diversity and MAF for each of the 21 chromosomes in the wheat genome

Chromosome	Number of marker	PIC^a^	Gene Diversity	MAF^b^
1A	361	0.2490	0.3042	0.2179
2A	641	0.2437	0.2938	0.2010
3A	205	0.2589	0.3174	0.2253
4A	403	0.2410	0.2925	0.2056
5A	184	0.2461	0.2998	0.2120
6A	444	0.2655	0.3272	0.2363
7A	339	0.2603	0.3211	0.2349
**A genome**	**2577**	**0.2521**	**0.3080**	**0.2190**
1B	578	0.2654	0.3283	0.2396
2B	764	0.2501	0.3061	0.2188
3B	831	0.2541	0.3105	0.2205
4B	300	0.2501	0.3058	0.2202
5B	563	0.2469	0.3003	0.2091
6B	302	0.2402	0.2898	0.1984
7B	317	0.2735	0.3393	0.2503
**B genome**	**3655**	**0.2543**	**0.3114**	**0.2224**
1D	204	0.2529	0.3112	0.2284
2D	315	0.2527	0.3094	0.2192
3D	130	0.2460	0.2988	0.2090
4D	102	0.2574	0.3147	0.2231
5D	255	0.2460	0.3002	0.2147
6D	290	0.2606	0.3193	0.2257
7D	371	0.2740	0.3415	0.2556
**D genome**	**1667**	**0.2557**	**0.3136**	**0.2251**
**Whole genome**	**7899**	**0.2540**	**0.3110**	**0.2222**

^a^ PIC, polymorphism information content

^b^ MAF, minor allele frequency

^c^ genome information were labeled in bold

### Linkage disequilibrium

**Fig. 3:**
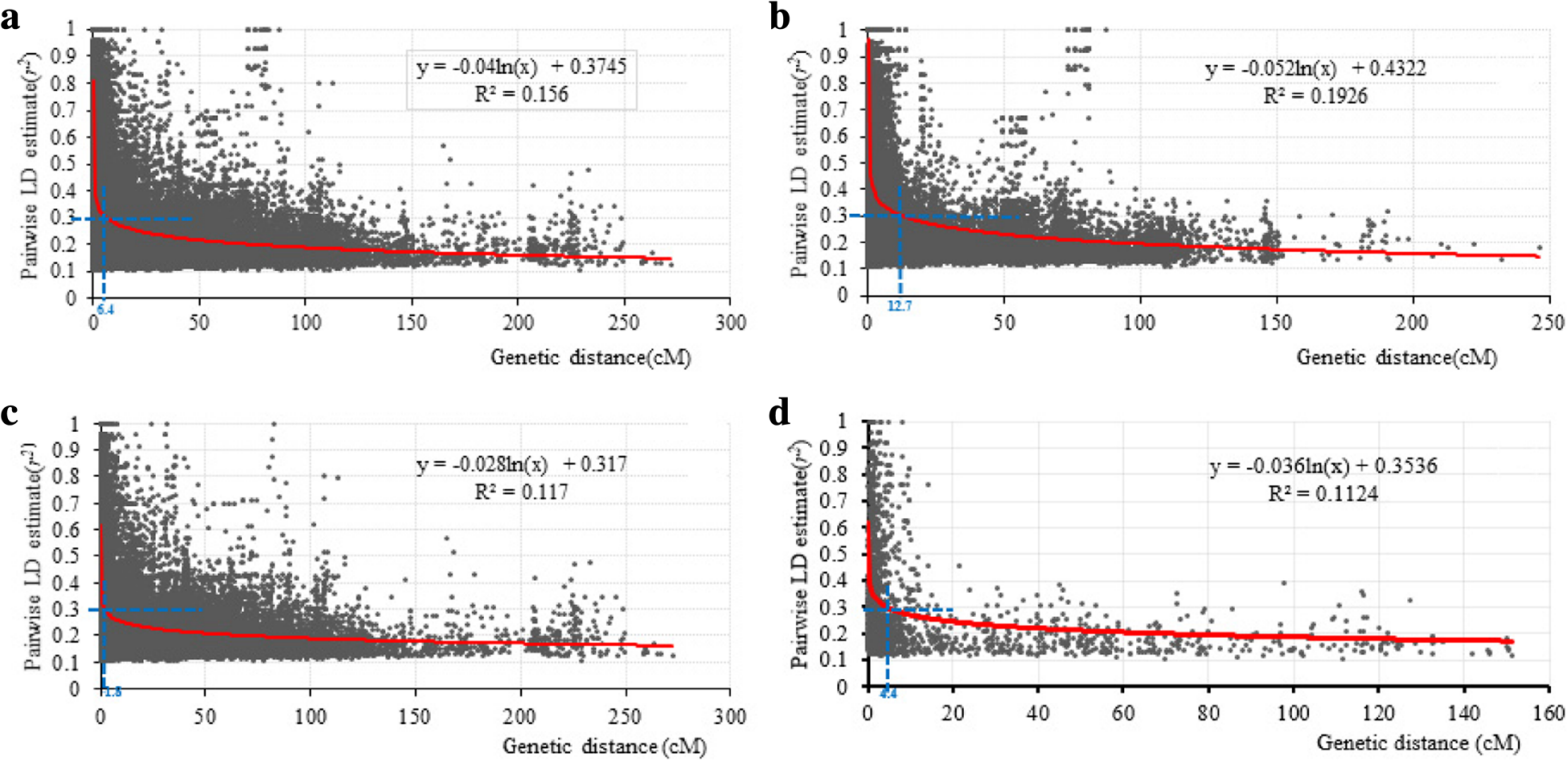
Linkage disequilibrium decay plot for 93 Northern Chinese wheat landraces based on 5486 DArT markers. The scatter plots showing pairwise DArT markers LD *r*^*2*^ value as a function of inter-marker genetic distances (cM). (**a**) Genome-wide average LD decay plot; (**b**) LD decay plot of A genome; (**c**) LD decay plot of B genome; (**d**) LD decay plot of D genome

Among the 7899 polymorphic markers, the map position of 5486 markers was known on the wheat consensus map version 4.0 (Additional file 3). These mapped markers (genome A = 1845 markers, B = 2871 markers, and D = 770 markers) were used to estimate LD values (Additional file 3). Scatter plots of LD values, represented as squared allele-frequency coefficients, between intra-chromosomal markers against the genetic distance are shown in Fig. 3. The fitted model suggested that LD decayed to *r*^*2*^ < 0.3 at 12.7 cM (Fig. 3b), 1.8 cM (Fig. 3c), and 4.4 cM (Fig. 3d) in the A, B, and D genomes, respectively. The LD decayed to the critical *r*^*2*^ value (0.30) for the entire genome at about 6.4 cM (Fig. 3a), which was used to determine the confidence interval for declaring distinct QTL. Thus, for markers that were significantly associated with stripe rust and located on the same chromosome, marker were considered to represent the same locus only if the genetic distance between the markers was less than 6.4 cM or the *r*^*2*^ value between the markers was greater than 0.3.

### GWAS of stripe rust resistance at the adult plant stage

**Fig. 4:**
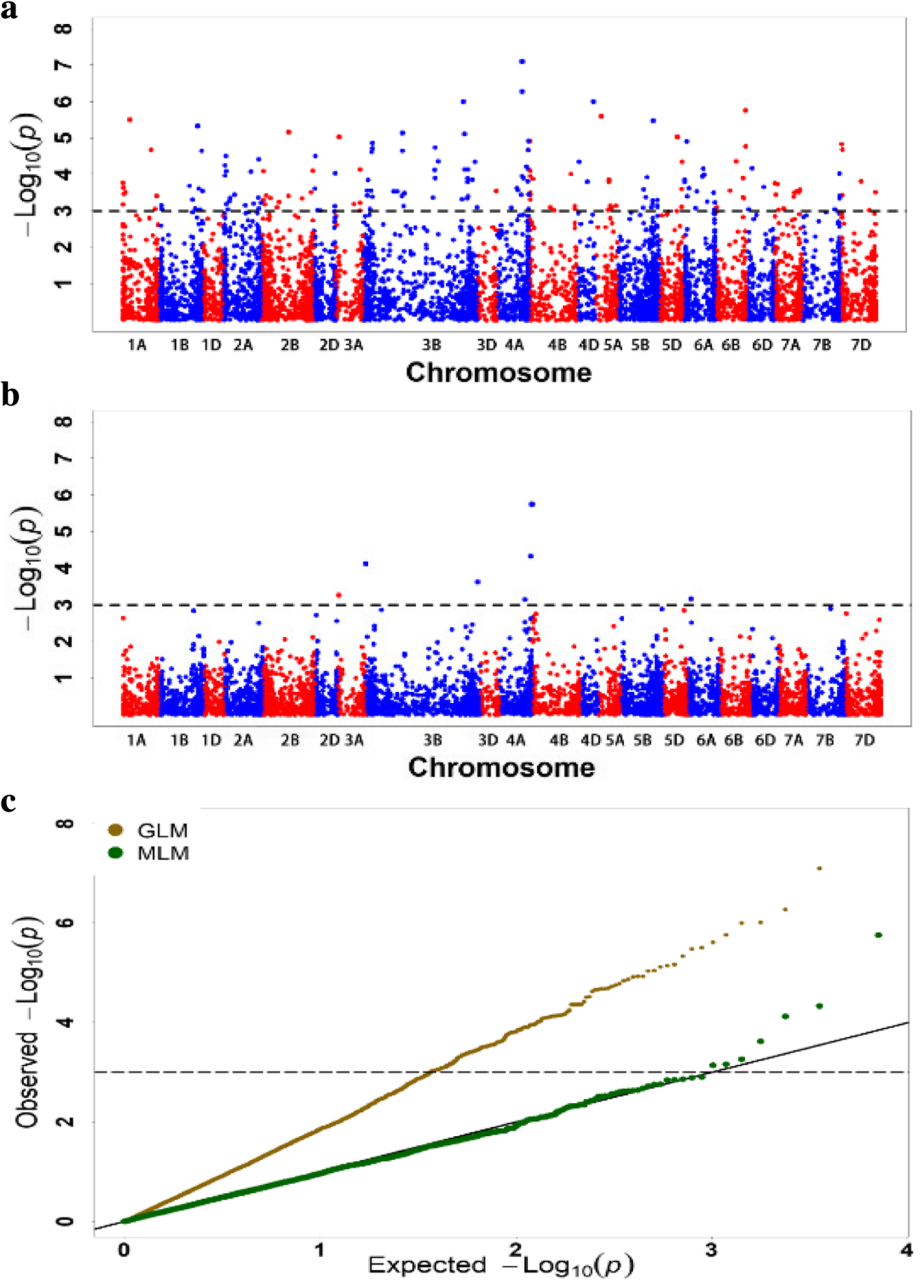
The GLM (**a**) and MLM (**b**) Manhattan plot of stripe rust resistance significantly associated markers. The horizontal line shows the genome-wide significant threshold *p* value of 0.001 or –log_10_ (*P*) value of 3.0. The A, B and D genomes are in red and blue colors successively. Q-Q plot (**c**) used to assess the fit of the model

Using the data for 7899 polymorphic SSR and DArT markers that had a missing data frequency less than 0.05 and MAF higher than 0.05, a GWAS was performed on IT and DS. The exploratory threshold for definition of marker-trait associations (MTAs) as significant was taken to be *P* < 0.001 (−log_10_ (*P*) > 3.00) [38]. To obtain reliable MTAs, phenotypic data collected from the four environments and BLUE_ALL were used. Using quantile-quantile (Q-Q) plots, we compared a general linear model (GLM) and MLM, both of which use the population structure or population structure and kinship as parameters for model calculation. The MLM method using a kinship matrix was the most efficient (Fig. 4). Association analyses between the two resistance traits (IT and DS) and the polymorphic markers showed that there were 32 significantly associated SSR and DArT markers (*P* < 0.001), among which 13 markers were significantly associated with IT and 19 markers were significantly associated with DS. Using the data recorded from the four environments and BLUE_ALL, 28 and 5 significantly associated markers were detected, respectively. Among the 28 markers, the majority (23) were detected at Mianyang in both years, and half (14) were detected in 2016 at the two locations (Table 3). The phenotypic explanation rates (*R*^2^) of these significant markers ranged from 12.63 to 34.36%. The associated loci were located on 13 chromosomes, namely 1B, 2D, 3A, 3B, 4A, 5A, 5B, 5D, 6A, 6B, 6D, 7B and 7D. Four markers were significantly associated with two different traits or environments, namely *990,726* was significantly associated with IT and DS in 2016 at Mianyang, *4406922* with IT in 2016 at Chongzhou and in 2016 at Mianyang, *995958* with DS in 2017 at Mianyang and BLUE_ALL of DS, and two allelic variant (*Xgwm169–4* and *Xgwm169–9*) of the SSR marker *Xgwm169* were significantly associated with DS in 2017 at Mianyang. The remainder of the markers was detected only in one environment (Table 3). Considering the LD decay distance observed in this study, significant markers within 6.4 cM were combined as a QTL, hence a total of 25 QTL regions were assigned based on IT and DS (Table 3). Although we developed the integrated map based on the linkage maps reported previously, a portion of the markers used in the present study could not be mapped because of the lack of sufficient common markers in the previous maps.

**Table 3:** Summary of quantitative trait loci for stripe rust resistance identified at the adult plant stage among 93 northern Chinese wheat landraces

QTL Name	Trait	Env.^a^	Marker	Position (cM)^b^	−log_10_(*P*)	R^2^	Reported QTL/genes^c^	Interval^d^	Reference
*QYr.sicau-1B*	DS	2016C	*1,254,926*	74.85	3	0.15	*Qyr.wpg-1B.2*	*IWA63-IWA2707*	Naruoka et al. [44]
							*QYr.cau-1BS*	*Xgwm264-Xwmc230*	Quan et al. [45]
*QYr.sicau-2D*	DS	2017 M	*1,112,673*	84.89	3.22	0.18	*QYr.caas-2DL*	*Xcfd44-wPt-6752*	Ren et al. [46]
							*QPst.jic-2D*	*Xgwm539-Xgwm349*	Melichar et al. [47]
*QYr.sicau-3A*	IT	2016 M	*1,131,042*	34.17	3.26	0.18		*wPt-6422-wPt-7890*	Rosewarne et al. [48]
*QYr.sicau-3B.1*	IT	2016 M	*7,491,774*	–	3.62	0.2	*–*	*–*	–
*QYr.sicau-3B.2*	IT	2016 M	*3,027,061*	9.02	4.13	0.24	*–*	*–*	–
*QYr.sicau-3B.3*	DS	2017 M	*1,201,570*	52.89	3.2	0.18	*–*	*–*	–
*QYr.sicau-4A.1*	DS&IT	2016 M	*990,726*	47.09	3.28	0.15	New		
		2016 M	*990,726*	47.09	3.14	0.14			
*QYr.sicau-4A.2*	IT	2016 M	*3,026,448*	119.65	4.33	0.25	*QYrst.orr-4AL*	*wPt-7924-rPt-7987*	Dolores et al. [49]
*QYr.sicau-4A.3*	IT	2016C	*4,406,922*	132.76	3.6	0.2	*QYrst.orr-4AL*	*wPt-9901-wPt-0032*	Dolores et al. [49]
	IT	2016 M	*4,406,922*	132.76	5.75	0.34	*QYrid.ui-4A*	*wPt-6603-wPt-2151*	Chen et al. [50]
							*QYr.sgi-4A.1*	*wPt-4620-Xwmc219*	Prins et al. [51]
*QYr.sicau-5A*	IT	2016C	*Xwmc410–3*	–	3.02	0.13	*QYr.caas-5AL*	*Xwmc410-Xbarc261*	Lan et al. [52]
*QYr.sicau-5B.1*	DS	2017C	*Xbarc172–7*	–	3.32	0.16	*–*	*–*	–
*QYr.sicau-5B.2*	DS	2017 M	*6,026,919*	–	3.09	0.17	*–*	*–*	–
*QYr.sicau-5B.3*	DS	2017 M	*3,533,933*	58.79	3.3	0.18	*QYrdr.wgp-5BL.1*	*iwa2365-iwa2093*	Hou et al. [53]
							*QYrdr.wgp-5BL.2*	*iwa3025-iwa5803*	Hou et al. [53]
							*QYr.sun-5B*	*wPt-3661-wPt-1733*	Bariana et al. [55]
							*QYrco.wpg-5B*	*iwa5488-wPt-6135*	Case et al. [54]
*QYr.sicau-5B.4*	DS	2017 M	*3,385,066*	–	3.26	0.18	*–*	*–*	–
*QYr.sicau-5B.5*	DS	2017 M	*1,270,827*	131.85	3.19	0.18	*QYrdr.wgp-5BL.1*	*iwa2365-iwa2093*	Hou et al. [53]
							*QYrdr.wgp-5BL.2*	*iwa3025-iwa5803*	Hou et al. [53]
*QYr.sicau-5D*	IT	BLUE_ALL	*1,099,801*	–	3.55	0.19	*–*	*–*	–
*QYr.sicau-6A.1*	IT	2016 M	*1,204,251*	36.52	3.16	0.18	New		
*QYr.sicau-6A.2*	DS	2017 M	*1,695,635*	87.39	3.83	0.21	New		
	DS	2017 M	*3,955,268*	87.60	3.29	0.18			
*QYr.sicau-6A.3*	DS	2017 M	*Xgwm169–4*	–	4.35	0.25	New		
	DS	2017 M	*Xgwm169–9*	–	3.15	0.17			
*QYr.sicau-6B*	DS	2017 M	*3,025,054*	31.19	3.34	0.18	*Yr78*	*Xwmc737-IWA7257*	Dong et al. [56]
	DS	2017 M	*1,074,322*	26.85	3.69	0.21	*QYrst.wgp-6BS.2*	*Xgwm132-Xgdm113*	Santra et al. [57]
*QYr.sicau-6D*	IT	2016C	*Xcfd95–2*	–	3.15	0.13	*QYr.ufs-6D*	*Xgwm325-Xbarc175*	Agenbag et al. [58]
							*YrH9020a*	*Xbarc202-Xbarc96*	Liu et al. [59]
*QYr.sicau-7B*	IT	BLUE_ALL	*5,010,940*	–	3.41	0.15	–	–	–
*QYr.sicau-7D.1*	DS	2017 M	*995,958*	65.83	4.12	0.23	New		
		BLUE_ALL	*995,958*	65.83	3.16	0.17			
*QYr.sicau-7D.2*	DS	2016 M	*1,095,389*	94.32	3.54	0.16	New		
			*993,762*	97.09	3.54	0.16			
*QYr.sicau-7D.3*	IT	BLUE_ALL	*4,004,033*	–	3.13	0.17	–	–	–

^a^ Four environments in this study, where 2016M = 2016 Mianyang, 2016C = 2016 Chongzhou, 2017M = 2017 Mianyang, 2017C = 2017 Chongzhou; BLUE_ALL was obtained across environments considering genotypes as a fixed effect in the model

^b^ The markers were positioned on the DArT-seq consensus map version 4.0 provided by Diversity Arrays Technology (https://www.diversityarrays.com)

^c^ *Yr* genes or QTL reported in previous studies, “-” indicates the QTL detected in the present study that could not be localized on the integrated map, “New” indicates the QTL detected in the present study that have not been reported preciously

^d^ The interval of the reported QTL or genes on the integrated map of previously reported stripe rust resistance genes and QTL (Additional file 6)

**Fig. 5:**
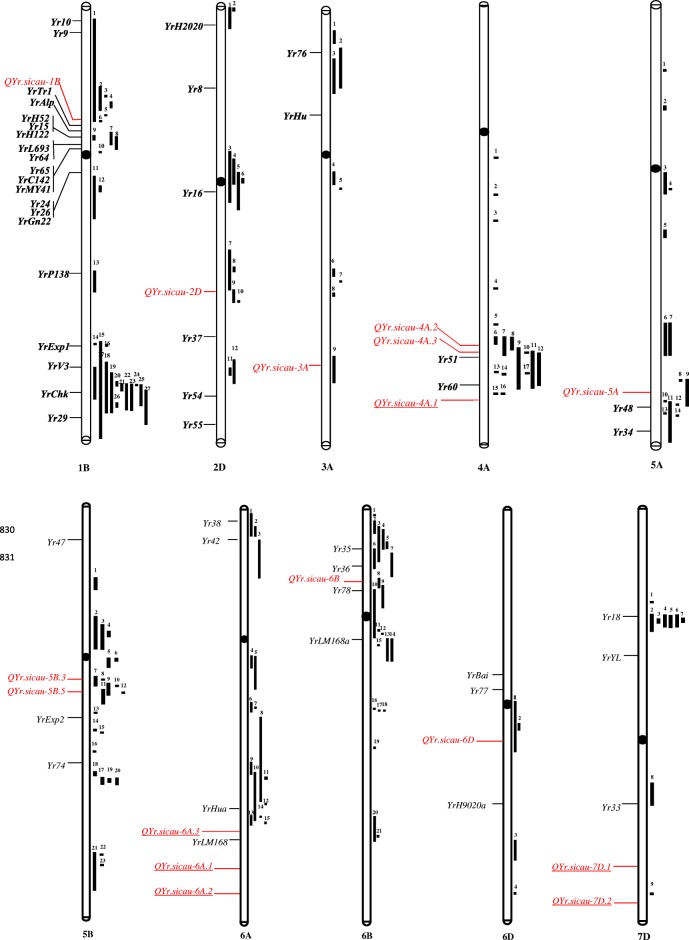
Chromosomal positions comparison between present detected *Pst* resistance QTL and reported *Yr* genes and QTL. All chromosomes are of standardized same relative lengths. QTL identified in this study and previously reported *Yr* genes are on left side of the chromosomes in red and black, respectively, and QTL for stripe rust resistance (black bar) are on right side of the chromosomes. The underlined QTL on the left side of the chromosomes might be novel loci in this study. All positions are approximations, and thus should be treated as guidelines for future studies. The detailed information of relationships between the QTL identified in this study and previously mapped *Yr* genes and QTL are based on this result integrated by the BioMercator V4.2 and described in the unpublished data of our research group (Table 3 and Additional file 6)

Of the 25 QTL, 16 with a genetic position on the integrated map were detailed as follows (Table 3). Among the 16 QTL, *QYr.sicau-1B*, *QYr.sicau-2D*, *QYr.sicau-3A*, *QYr.sicau-4A.2*, *QYr.sicau-4A.3*, *QYr.sicau-5A*, *QYr.sicau-5B.3*, *QYr.sicau-5B.5*, *QYr.sicau-6B* and *QYr.sicau-6D* were co-located with previously reported stripe rust resistance QTL and genes [44–59]. The remaining six QTL on chromosomes 4A (*QYr.sicau-4A.1*), 6A (*QYr.sicau-6A.1*, *QYr.sicau-6A.2* and *QYr.sicau-6A.3*) and 7D (*QYr.sicau-7D.1* and *QYr.sicau-7D.2*) were presumed to be newly identified when compared with the QTL and genes on the integrated map (Table 3, Fig. 5).

### Influence of favorable alleles number on response to *Pst*

**Fig. 6:**
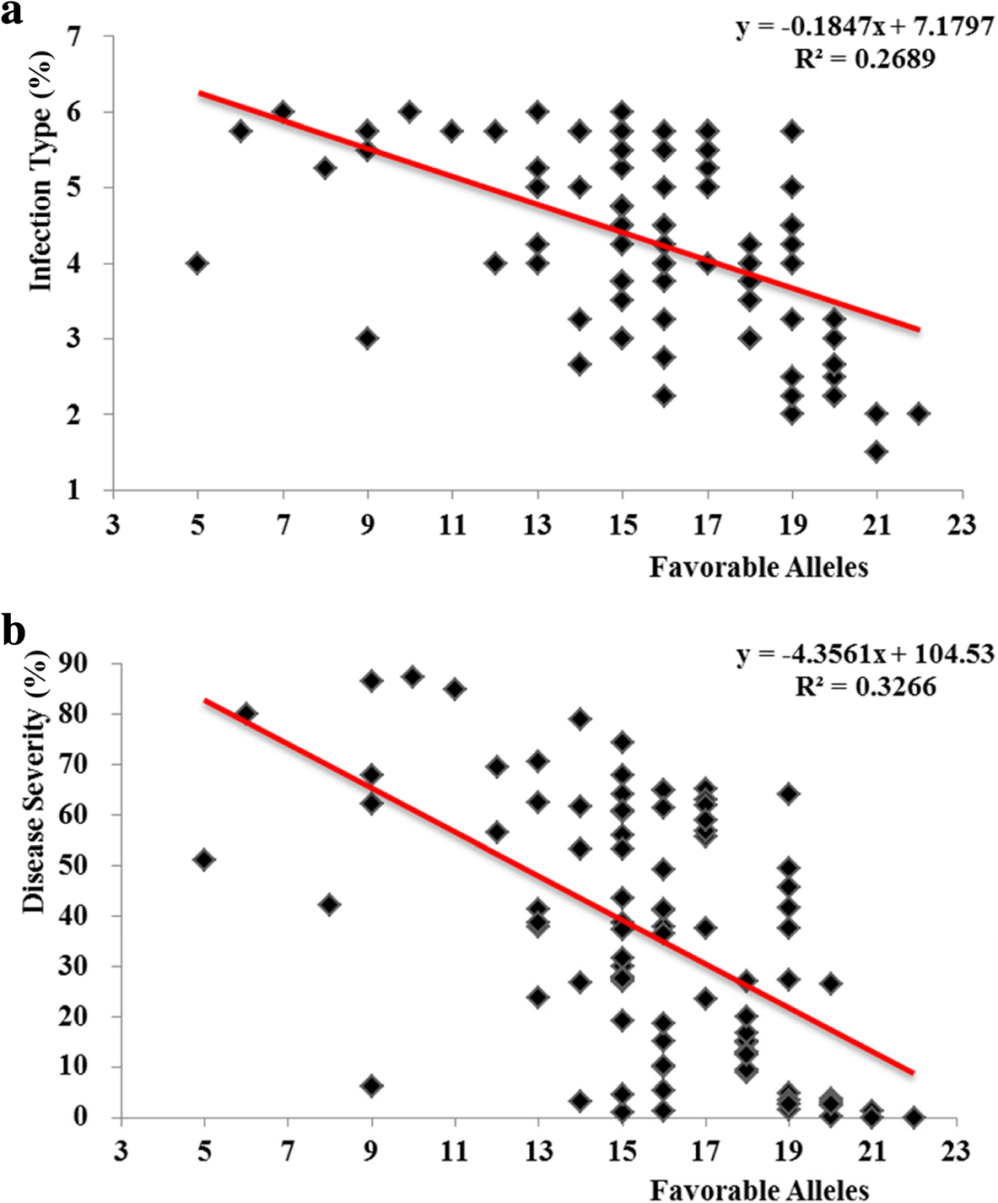
Regression of reaction to *Pst* against number of favorable alleles in the 93 accessions. (**a**) Infection type. (**b**) Disease severity. Original data are available in Additional file 5

A total of 32 significantly associated markers with reactions to *Pst* were identified. The number of favorable alleles among the 93 landraces ranged from 5 to 22. After ranking the accessions in increasing order based on the number of favorable alleles, comparison of the bottom 5% with a mean number of 7 favorable alleles to the top 5% (mean number 21.2 of favorable alleles) revealed that the former accessions showed significantly higher mean values of IT (4.73) and DS (50.93%), whereas the latter accessions showed lower mean values of IT (2.57) and DS (13.48%). The accessions that harbored relatively few of the identified resistance-associated favorable alleles showed a comparatively high disease index. Similar to a previous report by Maccaferri et al. [20], resistance to *Pst* was enhanced continuously with increase in the number of favorable alleles (Fig. 6), which revealed the additive effect of accumulation of alleles. The favorable alleles of *990,726*, *1,270,827*, *Xgwm169–4* and *995,958* were present in all 17 stable-resistance landraces, whereas the favorable alleles of *Xgwm169–9*, *Xcfd95–2*, *5,010,940* and *1,074,322* were only harbored in some of the 17 accessions with lower frequencies (6–18%), which revealed that *Pst* resistance in the landraces was polygenic (Additional file 5).

## Discussion

### Phenotypic variability and molecular diversity of the northern Chinese wheat landraces germplasm

The utilization of wheat landraces has gained increased interest in recent years. An enhanced understanding of the extent of genetic diversity and the genetic basis of responses to stripe rust in wheat may improve the effectiveness of exploration of genetic resources and enrich breeding for durable stripe rust resistance in wheat [60]. In the current study, 93 Northern Chinese wheat landraces were surveyed under four environments and challenged with a mixture of *Pst* isolates. The data revealed that the Northern Chinese landraces, in particular the 27 accessions grouped in subpopulation 2, possess abundant variation for resistance to stripe rust. The 17 accessions showed stable stripe rust resistance across four artificial inoculation environments, which indicated that these accessions carried genes effective against the *Pst* isolates prevalent in the study years and might be useful as parental breeding lines for improvement of stripe rust resistance in wheat. The remaining 76 accessions displayed high IT and DS in one environment, and low IT and DS in another environment (Fig. 1, Additional file 1). Variation in the prevalent pathogenic races in different trials and genotype by environment interactions may have led to difference in the number of resistant accessions across environments. Such findings have been reported in many previous studies [61, 62].

Genetic diversity is the probability of two randomly chosen alleles from the population being different; PIC estimates the detection power and informativeness of the molecular markers [63, 64]. The genome-wide average gene diversity values were 0.25 and 0.34 for subpopulations 1 and 2, respectively, and the PIC values were 0.20 and 0.27 for the respective subpopulations (Additional file 3). The values of both parameters were similar to those reported in previous studies [3], but also higher in subpopulation 2 than in subpopulation 1 in the present study. These results revealed the potential utility of the landraces for GWAS.

### Map-based comparison of significant stripe rust resistance loci with previously published *Yr* genes

To identify novel genes for effective resistance to *Pst* in China, we performed field evaluations at two locations in Sichuan with entirely different environments where stripe rust is endemic. The strong correlations among environments were also reflected in high heritability for IT (0.85) and DS (0.80) (Table 1), which supported the conclusion that the tested landraces were suitable for identification of significant associations in GWAS analyses. Accompanied with high heritability values, 32 significantly associated markers were detected at the exploratory threshold of *P* < 0.001 in this study (Table 3). Among the 32 significant markers, 28 were detected in the four environments and four in BLUE_ALL. However, the markers associated with each individual environment did not overlap with the BLUE_ALL associated markers. The results may reflect the small size of the study panel [65–69]. Considering the LD decay distance (6.4 cM) in the present study and marker position in the integrated map, a total of 16 QTL were detected (Table 3, Additional file 6).

The QTL *QYr.sicau-1B* was detected for DS at position 74.85 cM on chromosome 1B (Table 3). Twelve designated stripe rust resistance genes (*Yr10*, *Yr9*, *YrTr1*, *YrAlp*, *YrH52*, *Yr15*, *YrH122*, *YrL693*, *Yr64*, *Yr65*, *YrC142* and *YrMY41*) are located on the short arm of chromosome 1B. Several QTL for stripe rust resistance have also been detected on chromosome 1B, as shown on the integrated map (Fig. 5, Additional file 6). Notably, *QYr.sicau-1B* overlapped with *Qyr.wpg-1B.2* and *QYr.cau-1BS* reported in previous research [44, 45]. *QYr.cau-1BS* was associated with the stripe rust latent period and *Qyr.wpg-1B.2* showed a significant association with IT for three tested races.

The QTL *QYr.sicau-2D* was detected for DS at position 84.89 cM on chromosome 2D (Table 3). Compared with other chromosomes, fewer officially named *Yr* genes and QTL were detected, namely *YrH2020*, *Yr8*, *Yr16*, *Yr37*, *Yr54*, *Yr55* and several QTL (Fig. 5, Additional file 6). Among the loci detected, Ren et al. [46] identified a QTL (*QYr.caas-2DL*) in the Shanghai 3/Catbird (SHA3/CBRD) × Naxos F_6_ recombinant inbred line (RIL) population, which might be closely linked or identical to *QPst.jic-2D* identified in the UK winter wheat ‘Guardian’ [47]. On the integrated map used in the present study, *QYr.sicau-2D* overlapped with the former two QTL. Thus, they may represent the same or closely linked loci.

The QTL *QYr.sicau-3A* was detected for IT at position 34.17 cM on chromosome 3A (Table 3). *Yr76*, *YrHu* and several QTL were mapped on chromosome 3A (Fig. 5, Additional file 6). *QYr.sicau-3A* overlapped with the QTL derived from Avocet in the wheat ‘Avocet’ × ‘Pastor’ population [48].

Three QTL, namely *QYr.sicau-4A.1*, *QYr.sicau-4A.2* and *QYr.sicau-4A.3* were detected for IT and DS at position 47.09, 119.65 and 132.76 cM on chromosome 4A (Table 3). For *QYr.sicau-4A.1,* the marker *990,726* was simultaneously associated with IT and DS in 2016 at Mianyang. *QYr.sicau-4A.1* was located far from the reported genes *Yr51*, *Yr60* and QTL located on the integrated map of chromosome 4A (Fig. 5, Additional file 6), and thus it is likely to be a newly detected QTL. *QYr.sicau-4A.2* overlapped with *QYrst.orr-4AL* detected in an F_7_ RILs population derived from the cross between ‘Stephens’ and ‘Platte’ with the flanking markers *wPt-7924* and *rPt-7987* [49], which indicated that the two QTL are located in the same chromosome region. Similarly, three reported QTL (*QYrst.orr-4AL*, *QYr.sgi-4A.1* and *QYrid.ui-4A*) were also considered to be located in the same region as *QYr.sicau-4A.3* detected in the present study. *QYrst.orr-4AL* with different flanking markers *wPt-7924* and *rPt-7987* on the integrated map, overlapped with *QYr.sicau-4A.3*. A minor QTL *QYr.sgi-4A.1* was detected in several studies [50, 51] in a ‘Kariega’ × ‘Avocet S’ doubled haploid (DH) mapping population. The major QTL *QYrid.ui-4A* identified in the ‘Rio Blanco’ × ‘IDO444’ F_8:10_ RILs population shared the linked marker *Xgwm160* in common with *QYr.sgi-4A.1*.

The QTL *QYr.sicau-5A* was identified for IT on chromosome 5A (Table 3). The *Yr* genes and QTL reported on chromosome 5A may be located on the long arm, such as *Yr48*, *Yr34* and *QYr.caas-5AL*, as shown on the integrated map of chromosome 5A (Fig. 5, Additional file 6). *QYr.sicau-5A* was associated with the SSR marker *Xwmc410*, which is a flanking marker for *QYr.caas-5AL* [52]. Thus, it is likely that the two QTL were at the same locus.

*QYr.sicau-5B.3* and *QYr.sicau-5B.5* were detected for DS at position 58.79 and 131.85 cM on chromosome 5B (Table 3). As for other chromosomes in the B genome, many QTL and *Yr* genes for stripe rust have been detected on chromosome 5B, such as *Yr47*, *Yr74* and *YrExp2* (Fig. 5, Additional file 6). *QYr.sicau-5B.3* was located near to the previously reported QTL *QYrdr.wgp-5BL.1*, *QYrdr.wgp-5BL.2*, *QYrco.wpg-5B* and *QYr.sun-5B* [53–55]. Given that *QYrdr.wgp-5BL.2*, *QYrco.wpg-5B* and *QYr.sun-5B* were mapped for high-temperature adult-plant resistance, whereas *QYrdr.wgp-5BL.1* was mapped for race-specific all-stage resistance, these QTL certainly do not represent the same gene. In addition, in the present study, we only investigated the stripe rust response at the adult plant stage. Therefore, we cannot judge the resistance type of *QYr.sicau-5B.3* without further investigation. However, the positions of these QTL on chromosome 5B are likely to be adjacent. *QYr.sicau-5B.5* was also detected close to *QYrdr.wgp-5BL*.1 and *QYrdr.wgp-5BL.2.* The genetic distance between *QYr.sicau-5B.3* and *QYr.sicau-5B.5* on the V4.0 DArT consensus map (Table 3, Additional file 3) was beyond the confidence interval of the LD decay distance.

*QYr.sicau-6A.1* and *QYr.sicau-6A.2* were detected for IT and DS at positions 36.52 and 87.39–87.60 cM on chromosome 6A, while *QYr.sicau-6A.3* was detected with a SSR marker without a genetic distance on the DArT V4.0 consensus map (Table 3). Compared with the reported resistance genes *Yr38*, *Yr42*, *YrHua*, *YrLM168* and QTL on the integrated map of chromosome 6A, all three QTL might represent newly detected loci (Fig. 5, Additional file 6).

*QYr.sicau-6B* was detected with the DArT markers *3,025,054* and *1,074,322* for DS at positions 31.18 and 26.85cM on chromosome 6B (Table 3). The majority of *Yr* genes and QTL detected on chromosome 6B are located on the short arm, such as *Yr35*, *Yr36, Yr78* and QTL as displayed on the integrated map for 6B (Fig. 5, Additional file 6). *3,025,054* overlapped with *Yr78* [56] and *1,074,322* overlapped with *QYrst.wgp-6BS.2* on the consensus map [57]. *Yr78* was designated as synonymous with *QYr.wgp-6BS.1* which is close to the centromere of the 6BS chromosome but different from *QYrst.wgp-6BS.2* close to the telomere of chromosome 6B short arm [56, 57]. In the present study, by comparing the positions of the four QTL on the integrated map, *QYr.sicau-6B* was speculated to be synonymous with *Yr78* and *QYr.wgp-6BS.1*.

The QTL *QYr.sicau-6D* was detected for IT on chromosome 6D (Table 3). *YrBai*, *Yr77*, *YrH9020a* and several additional QTL were identified on chromosome 6D (Fig. 5, Additional file 6). *QYr.sicau-6D* overlapped with *QYr.ufs-6D* and *YrH9020a* on chromosome 6D [58, 59]. *QYr.ufs-6D* is associated with the leaf area infected phenotype [58] and is located between the SSR marker loci *Xgwm325* and *Xbarc175* on the long arm of chromosome 6D. The two closest linked flanking SSR markers (*Xbarc96* and *Xbarc202*) of *YrH9020a* are also located on the long arm of chromosome 6D.

The QTL *QYr.sicau-7D.1* and *QYr.sicau-7D.2* were detected for DS at position 65.83 and 94.32–97.09 cM on chromosome 7D (Table 3). As shown in the integrated map (Fig. 5), the majority of reported QTL on chromosome 7D, as well as *Yr18*, *Yr33* and *YrYL* were distributed on the short arm, except *Yr33*, which was located on the long arm (Additional file 6) and linked with the SSR markers *Xgwm437* and *Xgwm11* [70]. The closely linked markers *995,958*, *1,095,389* and *993,762* of the two QTL *QYr.sicau-7D.1* and *QYr.sicau-7D.2* were all located far from the reported loci. Therefore, it was speculated that they represent newly detected QTL for stripe rust.

The power of QTL detection by GWAS depends on sample size, number of markers, high LD and trait heritability [20, 62, 68]. The number of landraces (93) used in the current study was larger than that used for genome-wide association mapping of resistance to pre-harvest sprouting (80 Chinese wheat founder parents) [65], rust resistance mechanisms (33 orchardgrass accessions) [67], phenotypic traits (81 Canadian western spring wheat cultivars) [68], late maturity α-amylase activity (91 synthetic hexaploid wheat accessions) [69], and comparable to the 93 bread wheat accessions used for mapping agronomic traits [66]. However, the population size of the present study was smaller than that of several previous studies, which would lower the power of QTL detection. The landraces were highly diverse, as evidenced from the high rate of whole genome LD decay (6.4 cM, *r*^*2*^ = 0.30, Fig. 3a) with the 7899 DArT and SSR markers, and the average gene diversity and PIC of the whole genome were 0.3109 and 0.2540, respectively (Table 2). On the other hand, the 93 landraces represented the majority of the landraces collected by the Chinese Academy of Agricultural Sciences (CAAS) from the Northern Wheat-growing Zone in China. The landraces exhibited substantial and significant phenotypic variation in response to stripe rust (IT, 1.50–6.00; DS, 0–91%; Table 1, Fig. 1) among the four environments. The findings from the present research not only furnish valuable and practical information for acceleration of molecular breeding, but also provide novel sources of rust resistance for ongoing wheat improvement.

## Conclusion

Breeding for stripe rust resistance in modern wheat cultivars continues to be impeded by the narrow genetic basis of resistance in elite genetic backgrounds. Hence, wheat landraces, as an excellent genetic resource for bread wheat improvement, have attracted the attention of wheat researchers in recent years. The present study reports the presence of valuable genetic variation for multiple *Pst* races in the field among Northern Chinese wheat landraces. The landraces that showed stable resistance at the adult plant stage could be crossed with cultivars that exhibit desirable agronomic traits to enhance stripe rust resistance, particularly those accessions that harbor improved resistance alleles at novel loci. High-density, whole-genome DArT-seq markers revealed a high degree of genetic diversity and relatively rapid LD decay, which indicated that these 93 wheat landraces are suitable for GWAS to directly identify markers closely linked to the causal loci. In the GWAS analyses, 32 loci significantly associated with *Pst* resistance in the field were detected. Six QTL (*QYr.sicau-4A.1*, *QYr.sicau-6A.1*, *QYr.sicau-6A.2*, *QYr.sicau-6A.3*, *QYr.sicau-7D.1* and *QYr.sicau-7D.2*) were mapped to chromosomal regions in which no stripe rust resistance genes have been reported previously. This finding indicates that the landraces possess useful alleles currently underexploited in modern breeding germplasm, and that the landraces might carry novel resistance genes to stripe rust. However, allelism tests are required to confirm which of the identified QTL represent novel resistance genes and which represent alleles of previously mapped genes. The present results reveal the presence of novel *Pst* resistance loci in Northern Chinese Wheat Zone landraces that could be pyramided into common wheat cultivars by MAS, and provide closely linked markers to accelerate their validation and deployment in wheat breeding programs.

## Methods

### Plant materials

A total of 93 wheat landraces from the Northern Wheat-growing Zone in China were obtained from the CAAS. Accessions from eight provinces in the Northern Winter Wheat Zone (I) and Northern Spring Wheat Zone (VII) [41] in China were represented, including accessions from Inner Mongolia (12), Shanxi (21), Beijing (8), Shaanxi (29), Hebei (11), Gansu (6), Liaoning (3), and Tianjin (3) (Additional file 1).

### Phenotyping for stripe rust resistance at adult plant stage in field

The accessions were phenotyped for resistance to stripe rust at two field sites, Mianyang (31°48′ N, 104°73′ E) and Chongzhou (30°32′ N, 103°39′ E), in two growing seasons, 2015–2016 and 2016–2017, under an artificial inoculation environment. The different year-location combinations are referred to as “environments” and abbreviated as 2016 M, 2016C, 2017 M and 2017C, respectively. The accessions were sown in late October at Chongzhou and early November at Mianyang.

In all field trials, accessions in the stripe rust nurseries were evaluated as non-replicated three rows. Rows were 1.5 m long with 0.3 m spacing between rows and the susceptible check ‘Avocet S’, was planted every 20 rows. ‘SY95–71’, a highly susceptible wheat line, was planted as spreader rows bordering the nurseries to ensure production of sufficient inoculum to provide uniform stripe rust infection. At the fourth leaf stage, all seedlings of the spreaders and susceptible checks were artificially inoculated with a mixture of races prevalent in China, which included the officially named Chinese *Pst* races CYR31, CYR32, CYR33 and CYR34, and a series of pathotypes, for example, Guinong 22–14, Shui 4, and Shui 5, which were provided by the Plant Protection Institute of the Gansu Academy of Agricultural Sciences, Gansu, China. CYR34 shows the broadest spectrum of virulence in China, and it is avirulent to *Yr5*, *Yr8*, *Yr15*, *Yr24*, *Yr32*, and *YrTr1*, but is virulent to *Yr1*, *Yr2*, *Yr3*, *Yr4*, *Yr10*, *Yr25*, *Yr26*, *Yr44*, and *Yr76*, which are widely utilized in Chinese wheat cultivars [4, 71].

Stripe rust resistance was evaluated three times when disease severity on the flag leaves of the susceptible checks attained 60–100%. The stripe rust IT was estimated using a 0 to 4 scale (0, 0;, 1, 2, 3, 4) as described previously by Bariana and McIntosh [72] when the susceptible checks showed abundant sporulation. The scale values 0, 0;, 1, 2, 3 and 4 was converted to 1, 2, 3, 4, 5 and 6, respectively, prior to statistical analysis. Plants of IT 1–4 and of 5–6 were considered to be resistant and susceptible, respectively. Stripe rust DS was recorded weekly as the percentage leaf area with disease symptoms, from when the disease severity on the flag leaves of the susceptible checks attained 80–90% until after the peak severity. The DS thresholds of 0–20(%), 21–40(%) and 41–60(%) represented high, moderate and low levels of APR, respectively, whereas 61–80(%) and 81–100(%) indicated moderate and high susceptibility to stripe rust, respectively.

### Genotyping

Genomic DNA was extracted from young leaf tissue of 2-week-old seedlings using modified cetyltrimethyl ammonium bromide method as essentially described by Saghai Maroof et al. [73]. The DNA concentration was determined and diluted to a working solution of 50–100 ng/μL. The collection of 93 wheat landraces was genotyped using the DArT-seq (Diversity Arrays Technology, Canberra, ACT, Australia) genotyping-by-sequencing (GBS) platform. A total of 89,284 probes from the DArT-seq (DArT and DArT_GBS) were used for genotyping. The accessions were also screened with 450 SSR markers, which were obtained from GrainGenes database (http://wheat.pw.usda.gov) and those reported by Peng et al. [74], Suenaga et al. [75] and Li et al. [76]. PCR amplification of SSR markers was performed with the following thermal cycling conditions: initial denaturation at 94 °C for 5 min, followed by 35 cycles of denaturation at 94 °C for 40 s, annealing at 50–65 °C for 30 s depending on the primers, and extension at 72 °C for 1 min with final elongation of 7 min. After amplification, PCR products in a reaction volume of 3 μL were resolved by electrophoresis in 6% denaturing polyacrylamide gel and revealed by silver staining in according with the method of Bassam et al. [77]. The GWAS marker data were filtered based on the following criteria. Monomorphic markers and markers with maximum missing values of 5% were discarded and only those with MAF ≥ 0.05 were used for further analyses.

### Phenotypic data analysis

An analysis of variance (ANOVA) was used to test for additive variance between genotypes, environments, and the interaction between genotypes and environments using SAS V8.0 (SAS Institute, Cary, NC, USA). Broad-sense heritability (*H*^2^) was calculated using the ANOVA model to estimate the variance components on an accession mean basis. BLUE values were obtained across locations and years when considering genotypes as a fixed effect in the model using QTL IciMapping. BLUE values were also used to perform GWAS with the four environments resistance phenotype. The correlation between different environments was calculated with Spearman’s correlation coefficient.

### Genome-wide association study for stripe rust resistance

The association of the two marker sets (DArT and SSR markers) and stripe rust disease phenotype based on the adult stage in the field evaluation was carried out using a unified mixed linear model as implemented in TASSEL 3.0 software [78] (http://www.maizegenetics.net). PowerMarker V3.25 was used to estimate the genetic diversity of the DArT and SSR data [79]. The population structure of the 93 wheat landraces was assessed using the Bayesian clustering algorithm conducted with STRUCTURE V2.3.4 with a burn-in period at 50,000 iterations and a run of 100,000 replications of Markov Chain Monte Carlo (MCMC) after the burn in [80–82]. Manhattan plots were generated using the “Manhattan” function in the “qqman” package [83] in R × 64 3.4.3 (R Core Team, 2014). The Q-Q plots were used to assess the fit of the model.

### Comparison of significant resistance loci with previously reported *Yr* genes and QTL

For comparison with previous studies, we generated an integrated map of the stripe rust resistance genes and QTL reported previously (referred to herein as the integrated map), including 80 officially named *Yr* genes, 67 temporarily named *Yr* genes and 327 previously mapped QTL of different marker types [21, 84]. The map positions of the *Yr* gene or QTL in the integrated map were based on the ‘Synthetic’ × ‘Opata’ DH GBS map [85], the 9 K SNP consensus map [86], the sequential projection of the 90 K SNP consensus map [87], the tetraploid consensus map [88], the Diversity Array Technology consensus map V4.0 (http://www.diversityarrays.com), the 2004 SSR consensus map [89] and the ‘Synthetic’ × ‘Opata’ ITMI BARC SSR map [90]. The DArT and SSR markers significantly associated with stripe rust resistance in this study were mapped based on this integrated map using BioMercator V4.2.

## Additional files

Additional file 1: Ninety-three Northern Chinese wheat landrace accessions used in this study and AS_NO, ZM_NO, geographic origins, wheat plant zone in China, subpopulation (Q) and the phenotype of stripe rust (DS and IT). (XLSX 20 kb)

Additional file 2: Spearman’s correlations coefficients of response to stripe rust evaluated in four environments and BLUE_ALL. (DOCX 20 kb)

Additional file 3: Information of the 7899 DArT-seq markers and SSR markers used in this study. (XLSX 1270 kb)

Additional file 4: The genome specific comparisons of molecular diversity between subpopulation 1 and subpopulation 2 landraces. (DOCX 9540 kb)

Additional file 5: Distribution of resistance-associated alleles of significant markers (*P* < 0.001) in the diversity panel. (XLSX 23 kb)

Additional file 6: The details of QTL and *Yr* genes located on the integrated map. (XLSX 30 kb)

## Abbreviations

2016 M: 2016 Mianyang
2016C: 2016 Chongzhou
2017 M: 2017 Mianyang
2017C: 2017 Chongzhou
ANOVA: analysis of variance
APR: adult plant resistance
ASR: all stage resistance
BLUE: best linear unbiased estimators
CAAS: Chinese Academy of Agricultural Sciences
DArT: Diversity Arrays Technology
DH: doubled haploid
DS: disease severity
GBS: genotyping-by-sequencing
GLM: general linear model
GWAS: genome-wide association study
IT: infection type
I-zone: Northern Winter Wheat Zone
LD: linkage disequilibrium
MAF: Minor allele frequency
MAS: marker assisted selection
MLM: mixed linear model
MTAs: marker-trait associations
PIC: polymorphism information content
*Pst*: *Puccinia striiformis* f. sp. *tritici*
Q-Q plot: quantile-quantile plot
RIL: recombinant inbred line
SSR: simple sequence repeat
VII-zone: Northern Spring Wheat Zone

## References

[1] Schwessinger B (2017). Fundamental wheat stripe rust research in the 21^st^ century. New Phytol.

[2] Chen XM (2005). Epidemiology and control of stripe rust (*Puccinia striiformis f. sp. tritici*) on wheat. Can J Plant Pathol.

[3] Muleta KT, Rouse MN, Rynearson S, Chen X, Buta BG, Pumphrey MO (2017). Characterization of molecular diversity and genome-wide mapping of loci associated with resistance to stripe rust and stem rust in Ethiopian bread wheat accessions. BMC Plant Biol.

[4] Wang L, Zheng D, Zuo SX, Chen XM, Zhuang H, Huang LL, Kang ZS, Zhao J (2018). Inheritance and linkage of virulence genes in Chinese predominant race CYR32 of the wheat stripe rust pathogen *Puccinia striiformis* f. Sp. *tritici*. Frontiers in. Plant Sci.

[5] Chen WQ, Wu LR, Liu TG, Xu SC, Jin SL, Peng YL, Wang BT (2009). Race dynamics, diversity, and virulence evolution in *Puccinia striiformis* f. Sp. *tritici*, the causal agent of wheat stripe rust in China from 2003 to 2007. Plant Dis.

[6] Chen XM (2007). Challenges and solutions for stripe rust control in the United States. Aust J Agric Res.

[7] Chen XM (2014). Integration of cultivar resistance and fungicide application for control of wheat stripe rust. Can J Plant Pathol.

[8] Golegaonkar PG, Singh D, Park RF (2009). Evaluation of seedling and adult plant resistance to *Puccinia hordei* in barley. Euphytica..

[9] Bariana HS. DISEASES | breeding for disease resistance. Encyclopedia of Applied Plant Sciences. 2003:244–53.

[10] Rosewarne GM, Herrera-Foessel SA, Singh RP, Huerta-Espino J, Lan CX, He ZH (2013). Quantitative trait loci of stripe rust resistance in wheat. Theor Appl Genet.

[11] Chen XM (2013). Review article: high-temperature adult-plant resistance, key for sustainable control of stripe rust. Am J Plant Sci.

[12] Feng JY, Wang MN, See DR, Chao S, Zheng YL, Chen XM (2018). Characterization of novel gene *Yr79* and four additional QTL for all-stage and high-temperature adult-plant resistance to stripe rust in spring wheat PI 182103. Phytopathology..

[13] Nsabiyera V, Bariana HS, Qureshi N, Wong D, Hayden MJ, Bansal UK (2018). Characterisation and mapping of adult plant stripe rust resistance in wheat accession Aus27284. Theor Appl Genet.

[14] Chen XM, Moore M, Milus EA, Long DL, Line RF, Marshall D, Jackson L (2007). Wheat stripe rust epidemics and races of *Puccinia striiformis* f. Sp. *tritici* in the United States in 2000. Plant Dis.

[15] Wan AM, Zhao ZH, Chen XM, He ZH, Jin SL, Jia QZ, Yao G, Yang JX, Wang BT, Li GB, Bi YQ, Yuan ZY (2004). Wheat stripe rust epidemic and virulence of *Puccinia striiformis* f. Sp. *tritici* in China in 2002. Plant Dis.

[16] Wan AM, Chen XM, He ZH (2007). Wheat stripe rust in China. Aust J Agric Res.

[17] Zeven AC (2002). Traditional maintenance breeding of landraces: 2. Practical and theoretical considerations on maintenance of variation of landraces by farmers and gardeners. Euphytica..

[18] Mujeeb-Kazi A, Kazi AG, Dundas I, Rasheed A, Ogbonnaya F, Kishii M, Bonnett D, Wang RC, Xu S, Chen P (2013). Chapter four-genetic diversity for wheat improvement as a conduit to food security.

[19] Wulff BB, Moscou MJ (2014). Strategies for transferring resistance into wheat: from wide crosses to GM cassettes. Front Plant Sci.

[20] Maccaferri M, Zhang J, Bulli P, Abate Z, Chao S, Cantu D, Bossolini E, Chen X, Pumphrey M, Dubcovsky J (2015). A Genome-Wide Association Study of Resistance to Stripe Rust (*Puccinia striiformis* f. sp. *tritici*) in a Worldwide Collection of Hexaploid Spring Wheat (*Triticum aestivum* L.). G3 Genes Genomes Genetics.

[21] Chen XM, Kang ZS (2017). Stripe Rust.

[22] Bokore FE, Cuthbert RD, Knox RE, Randhawa HS, Hiebert CW, Depauw RM, Singh AK, Singh A, Sharpe AG, N Diaye A. (2017). Quantitative trait loci for resistance to stripe rust of wheat revealed using global field nurseries and opportunities for stacking resistance genes. Theor Appl Genet.

[23] Zhu CS, Gore M, Buckler ES, Yu JM (2008). Status and prospects of association mapping in plants. Plant Genome.

[24] Yue F, Lu Q, Zhai RR, Zhang MC, Xu Q, Yang YL, Shan W, Yuan XP, Yu HY, Wang YP (2016). Genome wide association mapping for grain shape traits in *indica* rice. Planta..

[25] Gyawali S, Chao S, Vaish SS, Singh SP, Rehman S, Vishwakarma SR, Verma RPS (2018). Genome wide association studies (GWAS) of spot blotch resistance at the seedling and the adult plant stages in a collection of spring barley. Mol Breed.

[26] Zhao Z, Zhang H, Fu Z, Chen H, Lin Y, Yan P, Li W, Xie H, Guo Z, Zhang X (2017). Genetic-based dissection of arsenic accumulation in maize using a genome-wide association analysis method. Plant Biotechnol J.

[27] Wang YY, Li YQ, Wu HY, Hu B, Zheng JJ, Zhai H, Lv SX, Liu XL, Chen X, Qiu HM (2018). Genotyping of soybean cultivars with medium-density array reveals the population structure and QTNs underlying maturity and seed traits. Front Plant Sci.

[28] Huang C, Nie XH, Shen C, You CY, Li W, Zhao WX, Zhang XL, Lin ZX (2017). Population structure and genetic basis of the agronomic traits of upland cotton in China revealed by a genome-wide association study using high-density SNPs. Plant Biotechnol J.

[29] Bekele WA, Wight CP, Chao S, Howarth CJ, Tinker NA (2018). Haplotype based genotyping-by-sequencing in oat genome research. Plant Biotechnol J.

[30] Bolibok-Brągoszewska H, Targońska M, Bolibok L, Kilian A, Rakoczy-Trojanowska M (2014). Genome-wide characterization of genetic diversity and population structure in Secale. BMC Plant Biol.

[31] Cericola F, Portis E, Lanteri S, Toppino L, Barchi L, Acciarri N, Pulcini L, Sala T, Rotino GL (2014). Linkage disequilibrium and genome-wide association analysis for anthocyanin pigmentation and fruit color in eggplant. BMC Genomics.

[32] Zhang J, Zhao JT, Liang Y, Zou ZR (2016). Genome-wide association-mapping for fruit quality traits in tomato. Euphytica..

[33] Fè D, Cericola F, Byrne S, Lenk I, Ashraf BH, Pedersen MG, Roulund N, Asp T, Janss L, Jensen CS (2015). Genomic dissection and prediction of heading date in perennial ryegrass. BMC Genomics.

[34] Jadhav AA, Rayate SJ, Mhase LB, Thudi M, Chitikineni A, Harer PN, Jadhav AS, Varshney RK, Kulwal PL (2015). Marker-trait association study for protein content in chickpea (*Cicer arietinum* L.). J Genet.

[35] Nicolas SD, Péros JP, Lacombe T, Launay A, Paslier MCL, Bérard A, Mangin B, Valière S, Martins F, Cunff LL (2016). Genetic diversity, linkage disequilibrium and power of a large grapevine (*Vitis vinifera* L) diversity panel newly designed for association studies. BMC Plant Biol.

[36] Gouy M, Rousselle Y, Chane AT, Anglade A, Royaert S, Nibouche S, Costet L (2015). Genome wide association mapping of agro-morphological and disease resistance traits in sugarcane. Euphytica..

[37] Liu S, Fan CC, Li JN, Cai GQ, Yang QY, Jian W, Yi XQ, Zhang CY, Zhou YM (2016). A genome-wide association study reveals novel elite allelic variations in seed oil content of *Brassica napus*. Theor Appl Genet.

[38] Valluru R, Reynolds MP, Davies WJ, Sukumaran S (2017). Phenotypic and genome-wide association analysis of spike ethylene in diverse wheat genotypes under heat stress. New Phytol.

[39] Liu J, Feng B, Xu ZB, Fan XL, Jiang F, Jin XF, Cao J, Wang F, Liu Q, Yang L (2018). A genome-wide association study of wheat yield and quality-related traits in Southwest China. Mol Breed.

[40] Liu K, Sun XX, Ning TY, Duan XX, Wang QL, Liu TT, An YL, Guan X, Tian JC, Chen JS (2018). Genetic dissection of wheat panicle traits using linkage analysis and a genome-wide association study. Theor Appl Genet.

[41] He ZH, Rajaram S, Xin ZY, Huang GZ (2001). A history of wheat breeding in China. J Comp Neurol.

[42] Evanno G, Regnaut S, Goudet J (2005). Detecting the number of clusters in individuals using the software STRUCTURE: a simlation study. Mol Ecol.

[43] Earl DA, Vonholdt BM (2012). STRUCTURE HARVESTER: a website and program for visualizing STRUCTURE output and implementing the Evanno method. Conserv Genet Resour.

[44] Naruoka Y, Garlandcampbell KA, Carter AH (2015). Genome-wide association mapping for stripe rust (*Puccinia striiformis* F. Sp. *tritici*) in US Pacific northwest winter wheat (*Triticum aestivum* L.). Theor Appl Genet.

[45] Quan W, Hou GL, Chen J, Du ZY, Lin F, Guo Y, Liu S, Zhang ZJ (2013). Mapping of QTL lengthening the latent period of *Puccinia striiformis* in winter wheat at the tillering growth stage. Eur J Plant Pathol.

[46] Ren Y, Li ZF, He ZH, Wu L, Bai B, Lan CX, Wang CF, Zhou G, Zhu HZ, Xia XC (2012). QTL mapping of adult-plant resistances to stripe rust and leaf rust in Chinese wheat cultivar Bainong 64. Theor Appl Genet.

[47] Melichar JPE, Berry S, Newell C, Maccormack R, Boyd LA (2008). QTL identification and microphenotype characterisation of the developmentally regulated yellow rust resistance in the UK wheat cultivar Guardian. Theor Appl Genet.

[48] Rosewarne GM, Singh RP, Huerta-Espino J, Herrera-Foessel SA, Forrest KL, Hayden MJ, Rebetzke GJ (2012). Analysis of leaf and stripe rust severities reveals pathotype changes and multiple minor QTL associated with resistance in an avocet × Pastor wheat population. Theor Appl Genet.

[49] Dolores VM, James PC, Rieralizarazu O, Chen X, Heesacker A, Ammar K, Crossa J, Mundt CC (2012). Genetic analysis of adult plant, quantitative resistance to stripe rust in wheat cultivar 'Stephens' in multi-environment trials. Theor Appl Genet.

[50] Chen J, Souza EJ, Guttieri MJ, Chen X, Xu S, Hole D, Zemetra R (2012). Genome-wide identification of QTL conferring high-temperature adult-plant (HTAP) resistance to stripe rust (*Puccinia striiformis* f. Sp. *tritici*) in wheat. Mol Breed.

[51] Prins R, Pretorius ZA, Bender CM, Lehmensiek A (2011). QTL mapping of stripe, leaf and stem rust resistance genes in a Kariega × avocet S doubled haploid wheat population. Mol Breed.

[52] Lan CX (2010). Identification of genomic regions controlling adult-plant stripe rust resistance in Chinese landrace Pingyuan 50 through bulked segregant analysis. Phytopathology..

[53] Hou L, Chen XM, Wang MN, See DR, Chao S, Bulli P, Jing JX (2015). Mapping a large number of QTL for durable resistance to stripe rust in winter wheat Druchamp using SSR and SNP markers. PLoS One.

[54] Case AJ, Naruoka Y, Chen X, Garland-Campbell KA, Zemetra RS, Carter AH (2014). Mapping stripe rust resistance in a Brundage × coda winter wheat recombinant inbred line population. PLoS One.

[55] Bariana HS, Bansal UK, Schmidt A, Lehmensiek A, Kaur J, Miah H, Howes N, Mcintyre CL (2010). Molecular mapping of adult plant stripe rust resistance in wheat and identification of pyramided QTL genotypes. Euphytica..

[56] Dong Z, Hegarty JM, Zhang J, Zhang W, Chao S, Chen X, Zhou Y, Dubcovsky J (2017). Validation and characterization of a QTL for adult plant resistance to stripe rust on wheat chromosome arm 6BS (*Yr78*). Theor Appl Genet.

[57] Santra DK, Chen XM, Santra M, Campbell KG, Kidwell KK (2008). Identification and mapping QTL for high-temperature adult-plant resistance to stripe rust in winter wheat (Triticum aestivum L.) cultivar 'Stephens'. Theor Appl Genet.

[58] Agenbag GM, Pretorius ZA, Boyd LA, Bender CM, Prins R (2012). Identification of adult plant resistance to stripe rust in the wheat cultivar Cappelle-Desprez. Theor Appl Genet.

[59] Liu ZG, Yao WY, Shen XX, Chao KX, Yu F, Min-Zhou LI, Wang BT, Qiang LI, Jing JX (2014). Molecular mapping of a stripe rust resistance gene *YrH9020a* transferred from Psathyrostachys huashanica Keng on wheat chromosome 6D. J Integr Agric.

[60] Manickavelu A, Joukhadar R, Jighly A, Lan C, Huerta-Espino J, Stanikzai AS, Kilian A, Singh RP, Ban T (2016). Genome wide association mapping of stripe rust resistance in afghan wheat landraces. Plant Sci.

[61] Pasam RK, Bansal U, Daetwyler HD, Forrest KL, Wong D, Petkowski J, Willey N, Randhawa M, Chhetri M, Miah H (2017). Detection and validation of genomic regions associated with resistance to rust diseases in a worldwide hexaploid wheat landrace collection using Bayes R and mixed linear model approaches. Theor Appl Genet.

[62] Liu WZ, Maccaferri M, Bulli P, Rynearson S, Tuberosa R, Chen XM, Pumphrey M (2016). Genome-wide association mapping for seedling and field resistance to *Puccinia striiformis* f. Sp. *tritici* in elite durum wheat. Theor Appl Genet.

[63] Weir BS (1996). Genetic data analysis 2: methods for discrete population genetic data.

[64] Botstein D, White RL, Skolnick M, Davis RW (1980). Construction of a genetic linkage map in man using restriction fragment length polymorphisms. Am J Hum Genet.

[65] Lin Y, Liu SH, Liu YX, Liu YJ, Chen GY, Xu J, Deng M, Jiang QT, Wei YM, Lu YL, Zheng YL (2017). Genome-wide association study of pre-harvest sprouting resistance in Chinese wheat founder parents. Genet Mol Biol.

[66] Mwadzingeni L, Shimelis H, Rees DJ, Tsilo TJ (2017). Genome-wide association analysis of agronomic traits in wheat under drought-stressed and non-stressed conditions. PLoS One.

[67] Zeng B, Yan HD, Liu XC, Zang WJ, Zhang AL, Zhou SF, Huang LK, Liu JP (2017). Genome-wide association study of rust traits in orchardgrass using SLAF-seq technology. Hereditas..

[68] Chen H, Semagn K, Iqbal M, Moakhar NP, Haile T, N Diaye A, Yang RC, Hucl P, Pozniak C, Spaner D (2017). Genome-wide association mapping of genomic regions associated with phenotypic traits in Canadian western spring wheat. Mol Breeding.

[69] Emebiri LC, Oliver JR, Mrva K, Mares D (2010). Association mapping of late maturity α-amylase (LMA) activity in a collection of synthetic hexaploid wheat. Mol Breed.

[70] Zahravi M, Bariana HS, Shariflou MR, Balakrishma PV, Banks PM, Ghannadha MR (2003). Bulk segregant analysis of stripe rust resistance in wheat (*Triticum aestivum*) using microsatellite markers.

[71] Liu B, Liu TG, Zhang ZY, Jia QZ, Wang BT, Gao L, Peng YL, Jin SL, Chen WQ (2017). Discovery and pathogenicity of CYR34, a new race of *Puccinia striiformis* f. sp. *tritici* in China. Acta Phytopathologica Sinica.

[72] Bariana HS, Mcintosh RA (1993). Cytogenetic studies in wheat. XV. Location of rust resistance genes in VPM1 and their genetic linkage with other disease resistance genes in chromosome 2A. Genome..

[73] Saghai Maroof MA, Soliman KM, Jorgensen RA, Allard RW (1984). Ribosomal DNA spacer-length polymorphisms in barley: mendelian inheritance, chromosomal location, and population dynamics. PNAS..

[74] Peng JH, Fahima T, Röder MS, Huang QY, Dahan A, Li YC, Grama A, Nevo E (2000). High-density molecular map of chromosome region harboring stripe-rust resistance genes *YrH52* and *Yr15* derived from wild emmer wheat, *Triticum dicoccoides*. Genetica.

[75] Suenaga K, Singh RP, Huertaespino J, William HM (2003). Microsatellite markers for genes *lr34*/*yr18* and other quantitative trait loci for leaf rust and stripe rust resistance in bread wheat. Phytopathology..

[76] Li GQ, Li ZF, Yang WY, Zhang Y, He ZH, Xu SC, Singh RP, Qu YY, Xia XC (2006). Molecular mapping of stripe rust resistance gene *YrCH42* in Chinese wheat cultivar Chuanmai 42 and its allelism with *Yr24* and *Yr26*. Theor Appl Genet.

[77] Bassam BJ, Caetano-Anollés G (1993). Silver staining of DNA in polyacrylamide gels. Appl Biochem Biotechnol.

[78] Bradbury PJ, Zhang Z, Kroon DE, Casstevens TM, Ramdoss Y, Buckler ES (2007). TASSEL: software for association mapping of complex traits in diverse samples. Bioinformatics..

[79] Liu K, Muse SV (2005). PowerMarker: an integrated analysis environment for genetic marker analysis. Bioinformatics..

[80] Falush D, Stephens M, Pritchard JK (2003). Inference of population structure using multilocus genotype data: linked loci and correlated allele frequencies. Genetics..

[81] Hubisz MJ, Falush D, Stephens M, Pritchard JK (2010). Inferring weak population structure with the assistance of sample group information. Mol Ecol Resour.

[82] Pritchard JK, Stephens M, Rosenberg NA, Donnelly P (2000). Association mapping in structured populations. Am J Hum Genet.

[83] Turner SD (2018). qqman: an R package for visualizing GWAS results using Q-Q and manhattan plots. Biorxiv.

[84] Cheng YK, Yao FJ, Ye XL, Jiang QT, Li W, Deng M, Wei YM, Chen GY. Construction of linkage map of the meta quantitative trait loci (MQTL) on stripe rust resistance in Wheat (*Triticum aestivum* L.). Acta Phytopathologica Sinica. 2018. 10.13926/j.cnki.apps.000292.

[85] Cyrille S, Jiang D, Wang S, Eduard A (2013). Sequence-based mapping of the polyploid wheat genome. G3 Genes Genomes Genetics.

[86] Cavanagh CR, Chao S, Wang S, Huang BE, Stephen S, Kiani S, Forrest K, Saintenac C, Brownguedira GL, Akhunova A (2013). Genome-wide comparative diversity uncovers multiple targets of selection for improvement in hexaploid wheat landraces and cultivars. PNAS..

[87] Wang S, Wong D, Forrest K, Allen A, Chao S, Huang BE, Maccaferri M, Salvi S, Milner SG, Cattivelli L (2014). Characterization of polyploid wheat genomic diversity using a high-density 90,000 single nucleotide polymorphism array. Plant Biotechnol J.

[88] Maccaferri M, Ricci A, Salvi S, Milner SG, Noli E, Martelli PL, Casadio R, Akhunov E, Scalabrin S, Vendramin V (2015). A high-density, SNP-based consensus map of tetraploid wheat as a bridge to integrate durum and bread wheat genomics and breeding. Plant Biotechnol.

[89] Somers DJ, Isaac P, Edwards K (2004). A high-density microsatellite consensus map for bread wheat (*Triticum aestivum* L.). Theor Appl Genet.

[90] Song QJ, Shi JR, Singh S, Fickus EW, Costa JM, Lewis J, Gill BS, Ward R, Cregan PB (2005). Development and mapping of microsatellite (SSR) markers in wheat. Theor Appl Genet.

