# Supplementary material for: Characterization of molecular diversity and genome-wide association study of stripe rust resistance at the adult plant stage in Northern Chinese wheat landraces
Source: BMC Genet. 2019 Mar 26;20:38. doi: 10.1186/s12863-019-0736-x (PMC6434810; doi:10.1186/s12863-019-0736-x)
Supplement: Supplementary file 2 — Spearman’s correlations coefficients of response to stripe rust evaluated in four environments and BLUE_ALL. (DOCX 20 kb) [file 12863_2019_736_MOESM2_ESM.docx]

**Additional file 1** Spearman's correlations coefficients of response to stripe rust evaluated in four environments and BLUE_ALL

|  | 2016M | 2016C | 2017M | 2017C | BLUE_ALL |
| --- | --- | --- | --- | --- | --- |
| **IT vs. IT^a^** |  |  |  |  |  |
| 2016M | 1 |  |  |  |  |
| 2016C | 0.594^**^ | 1 |  |  |  |
| 2017M | 0.632^**^ | 0.594^**^ | 1 |  |  |
| 2017C | 0.537^**^ | 0.576^**^ | 0.748^**^ | 1 |  |
| BLUE_ALL | 0.771^**^ | 0.778^**^ | 0.906^**^ | 0.881^**^ | 1 |
| **DS vs. DS^b^** |  |  |  |  |  |
| 2016M | 1 |  |  |  |  |
| 2016C | 0.600^**^ | 1 |  |  |  |
| 2017M | 0.747^**^ | 0.478^**^ | 1 |  |  |
| 2017C | 0.635^**^ | 0.658^**^ | 0.664^**^ | 1 |  |
| BLUE_ALL | 0.882^**^ | 0.801^**^ | 0.813^**^ | 0.853^**^ | 1 |
| **IT vs. DS^c^** |  |  |  |  |  |
| 2016M | **0.867^**d^** | 0.656^**^ | 0.688^**^ | 0.600^**^ | 0.802^**^ |
| 2016C | 0.554^**^ | **0.864^**^** | 0.503^**^ | 0.587^**^ | 0.704^**^ |
| 2017M | 0.685^**^ | 0.570^**^ | **0.822^**^** | 0.648^**^ | 0.810^**^ |
| 2017C | 0.578^**^ | 0.634^**^ | 0.773^**^ | **0.958^**^** | 0.897^**^ |
| BLUE_ALL | 0.782^**^ | 0.772^**^ | 0.791^**^ | 0.820^**^ | **0.932^**^** |

^a^Comparisons between the infection type of different environments. 2016C = 2016 Chongzhou, 2016M = 2016 Mianyang, 2017C = 2017 Chongzhou, 2017M = 2017 Mianyang; BLUE-ALL were obtained across environments considering genotypes as a fixed effect in the model.

^b^Comparisons between the disease severity of different environments.

^c^Comparisons between the infection type and disease severity of different environments.

^d^Correlations coefficients between IT and DS of the same environment were labeled in bold and underlined. The P values of all the Spearman’s correlation coefficients in the table are smaller than 0.001 (*P* < 0.001)
