# Supplementary material for: Characterization of molecular diversity and genome-wide association study of stripe rust resistance at the adult plant stage in Northern Chinese wheat landraces
Source: BMC Genet. 2019 Mar 26;20:38. doi: 10.1186/s12863-019-0736-x (PMC6434810; doi:10.1186/s12863-019-0736-x)
Supplement: Supplementary file 4 — The genome specific comparisons of molecular diversity between subpopulation 1 and subpopulation 2 landraces. (DOCX 9540 kb) [file 12863_2019_736_MOESM4_ESM.docx]

**Additional file 4 The genome specific comparisons of molecular diversity between subpopulation 1 and subpopulation 2 landraces.** Gene Diversity and polymorphism information content (PIC) values were used to compare the extent of genetic variation in 93 Chinese Northern landraces. The blue bars are molecular diversity of subpopulation 1 and the red bars are that of subpopulation 2
